# Differences in Trapezius Muscle H-Reflex between Asymptomatic Subjects and Symptomatic Shoulder Pain Subjects

**DOI:** 10.3390/s23094217

**Published:** 2023-04-23

**Authors:** Ana S. C. Melo, Janet L. Taylor, Ricardo Ferreira, Bruno Cunha, Manuel Ascenção, Mathieu Fernandes, Vítor Sousa, Eduardo B. Cruz, J. Paulo Vilas-Boas, Andreia S. P. Sousa

**Affiliations:** 1 Center for Rehabilitation Research, ESS (Escola Superior de Saúde), Polytechnic of Porto, Rua Dr. António Bernardino de Almeida, 400, 4200-072 Porto, Portugal; 2 Research Centre in Physical Activity, Health and Leisure, Faculty of Sport, University of Porto, Rua Dr. Plácido Costa, 91, 4200-450 Porto, Portugal; 3 Porto Biomechanics Laboratory (LABIOMEP-UP), University of Porto, Rua Dr. Plácido Costa, 91, 4200-450 Porto, Portugal; 4 Center for Interdisciplinary Applied Research in Health, School of Health, Setubal Polytechnic Institute, Campus do IPS Estefanilha, 2914-503 Setubal, Portugal; 5 Centre for Human Performance, School of Medical and Health Sciences, Edith Cowan University, Perth, WA 6027, Australia; 6 Neuroscience Research Australia, Sydney, NSW 2031, Australia; 7 Department of Physiotherapy, Escola Superior de Saúde, Instituto Politécnico de Setúbal, Campus do IPS Estefanilha, 2914-503 Setúbal, Portugal; 8 Comprehensive Health Research Center (CHRC), Universidade Nova de Lisboa, 1169-056 Lisboa, Portugal; 9 Centre of Research, Education, Innovation and Intervention in Sport (CIFI2D), Faculty of Sport, University of Porto, Rua Dr. Plácido Costa, 91, 4200-450 Porto, Portugal

**Keywords:** shoulder pain, Hoffmann reflex, motoneuron recruitment, trapezius muscle, scapular stability, feedback mechanisms

## Abstract

In chronic shoulder pain, adaptations in the nervous system such as in motoneuron excitability, could contribute to impairments in scapular muscles, perpetuation and recurrence of pain and reduced improvements during rehabilitation. The present cross-sectional study aims to compare trapezius neural excitability between symptomatic and asymptomatic subjects. In 12 participants with chronic shoulder pain (symptomatic group) and 12 without shoulder pain (asymptomatic group), the H reflex was evoked in all trapezius muscle parts, through C3/4 nerve stimulation, and the M-wave through accessory nerve stimulation. The current intensity to evoke the maximum H reflex, the latency and the maximum peak-to-peak amplitude of both the H reflex and M-wave, as well as the ratio between these two variables, were calculated. The percentage of responses was considered. Overall, M-waves were elicited in most participants, while the H reflex was elicited only in 58–75% or in 42–58% of the asymptomatic and symptomatic participants, respectively. A comparison between groups revealed that the symptomatic group presented a smaller maximum H reflex as a percentage of M-wave from upper trapezius and longer maximal H reflex latency from the lower trapezius (*p* < 0.05). Subjects with chronic shoulder pain present changes in trapezius H reflex parameters, highlighting the need to consider trapezius neuromuscular control in these individuals’ rehabilitation.

## 1. Introduction

The nervous system (NS) acts through the relationship between excitation and inhibition [1], which could be altered in conditions such as pain [2]. In acute musculoskeletal pain conditions, the presence in the muscle of substances such as bradykinin, substance P or serotonin [3] leads to reduced nociceptor thresholds [4] as a protective response against injury [4,5]. However, with time, the repeated discharge of the peripheral nociceptors [6] and an impaired balance between descending inhibitory mechanisms and the pain facilitator pathways can lead to changes in the central modulation of sensory input from the muscle [2,3]. Thus, increased central excitability [5] of dorsal horn neurons [3] as well as enhanced response to inputs (hypersensitivity), known as central sensitization [2], may be experienced by some subjects with chronic pain, such as shoulder pain [5]. At the same time, an adaptation of the motor strategy to protect the painful area [7,8] and maintain the motor output [8], could lead to changes at the muscular level [3,4,5,7,8]. Both painful and non-affected muscles seem to change their mechanical behavior and activity [8,9]. These adaptations to pain are heterogeneous between subjects [10], vary with tasks and muscles [7,8] and their protective action could lead to consequences such as, for example, increased load to non-painful structures and decreased movement and variability [7].

Shoulder pain conditions stand out for their high prevalence in the general population, their prolonged recovery, and associated recurrences [11,12]. Some muscular changes [7,13] have been related to CNS adaptation mechanisms [14]. The adaptations in muscular mechanical behavior [8], together with possible changes in motoneuron or cortical excitability [7] and sensitization [2,3,4,5], might explain the maintained shoulder pain condition [2] and the high prevalence of pain recurrences in shoulder non-degenerative conditions [15] and could be the reason why rehabilitation is not always effective [10] or quick [2]. Beyond the alterations in the rotator cuff and other scapular musculature, previous studies have reported changes in trapezius muscle activity in subjects with maintained or recurrent shoulder pain. These changes include inhibition and/or changed timing of activation [16] of the lower (LT) and middle (MT) portions of trapezius, together with inconsistent findings regarding the upper portion (UT) [17,18]. Thus, for UT, some studies reported excessive activation [19,20,21,22,23] while others reported decreased UT activation [17,18,24]. These opposite findings regarding UT could possibly be related to two conditions. Some authors believe that increased UT activity could be related to the pain and the trigger points usually found over it [25], or to a compensatory strategy to elevate the arm during shoulder pain through increased clavicular elevation and, consequently, scapular elevation [21]. By contrast, other authors have associated impaired UT activity with the scapular dyskinesis presentation, namely scapular depression and upward rotation [18]. Nevertheless, it is important to mention that although this tonic scapular stabilizer [24] is considered by some authors to be responsible for scapular upward rotation, together with serratus anterior [18,26], the main role of UT is in clavicular motions (rotation, retraction and elevation). Its influence on the scapula occurs as a consequence, contributing in this way to scapula elevation and only for approximately 25% of upward rotation [27,28]. In addition to the mentioned adaptations, a previous study [29] also reported changes in a long-latency trapezius muscle reflex when comparing UT and LT of subjects with shoulder instability with healthy subjects. More specifically, in the case of shoulder instability, LT showed delayed or absent reflexes evoked from the afferents of the forearm and hand, which could reflect an impaired LT contribution to scapular stability during the use of the arm and hand [29].

Considering these facts, and remembering that muscular activity is exposed to the descending and reflex controls [30] that contribute to the scapular stability and movement [29], and that could be altered with shoulder pain, studies regarding muscular modulation in other shoulder pain conditions may add helpful knowledge [31] to improve the rehabilitation process [29]. The H reflex neural circuit occurs via a mainly monosynaptic connection in the spinal cord, whereby electrical stimulation of the group Ia afferents leads to an excitatory volley onto the motoneurons, evoking motoneuron depolarization/excitation and a consequent activation of the muscle fibers [32,33]. Thus, it can evaluate the modulation of monosynaptic reflex activity in the spinal cord [31,34,35] by assessing motoneuron excitability [31,34,35], independently of the muscle mechanoreceptors [33]. H reflex assessment could then be a useful tool to identify adaptations in the function of spinal structures following pain conditions or also therapeutic interventions [33]. Previous studies involving painful conditions in other body regions have found alterations in H reflexes. Painful areas in the gastrocnemius were associated with a decreased threshold and increased amplitude of H reflexes [36] but reductions in H reflex amplitude [9,37], as well as increases in threshold [37,38] and latency [37], occurred in the vastus medialis in patellofemoral pain [9] and soleus [37,38] in chronic low back pain and lumbosacral radiculopathy [37,38].

Unusually, the trapezius innervation is split between the C3/4 nerve (sensory information) and the accessory nerve (motor component) [13,31,39,40]. Thus, the amplitude of the maximum H reflex (evoked through stimulation of the C3/4 nerve) is little influenced by the M-wave [13], as the absence of motor axon direct activation [39] avoids collision between action potentials of the evoked reflex responses and the antidromic impulses evoked from the motor axon stimulation [13,31]. Despite the mentioned facts and the changes related to shoulder pain, the H reflex of the trapezius muscle [13,30,31,39,41] has not been studied in people with shoulder pain.

The present study, by investigating a neurophysiologic variable possibly related to shoulder pain adaptations, aimed to assess the possible differences in trapezius neural excitability between symptomatic and asymptomatic subjects. The reduction in H reflexes in other painful conditions [9,36,37,38] supports the hypothesis of a decreased H reflex response, with increased threshold and latency in symptomatic subjects. The results of the present study could be helpful for the rehabilitation field by improving knowledge regarding motor control changes in pain conditions, but could also be useful for technological development regarding H reflex assessment.

## 2. Materials and Methods

### 2.1. Subjects

Twenty-four volunteers (ten male and fourteen female) took part in this study. Participants, recruited from a School of Health via personal contact or email, were included in the symptomatic or asymptomatic groups, according to the eligibility criteria.

While the asymptomatic group included healthy subjects that had had no shoulder pain events in the last 2 years, the symptomatic group included subjects that presented continuous or intermittent (2 or more episodes in the last 3 months) chronic shoulder pain. Chronic shoulder pain fitted the criteria of: (a) pain in the upper arm, specifically in shoulder, deltoid and/or scapular areas; (b) non-specific or associated with a diagnosis (except if mentioned in the exclusion criteria); and (c) lasting more than 3 months. Subjects with a history of shoulder fracture, dislocation, tears, infection or neoplasm; shoulder surgery; cervical and/or thoracic pathologies or pain associated with active movements of these regions; neurological disease; and/or body mass index outside the range 18.5–30 kg/m^2^ [42,43,44] were excluded.

Subjects of the two groups were matched regarding gender, age, and dominant upper limb. Before participation, all subjects read and signed the informed consent form, and the study was approved by the local ethical committee (CE0071A).

### 2.2. Instrumentation

To characterize the participants, two self-reported questionnaires were applied to the symptomatic group and the scapular dyskinesis classification test was applied to all the subjects. The numeric rating scale (NRS), which, according to a previous study [45] has an intra-rater intraclass correlation coefficient (ICC) of 0.84, was used to quantify the pain intensity from 0 (“no pain”) to 10 (“unbearable pain”) [46]. The Portuguese version [47,48] of Shoulder Pain and Disability Index (SPADI), with an internal consistency reliability of α = 0.75 (for pain) and 0.84 (for functional activity), was used to characterize the shoulder function from 0 (“no pain/no difficulty”) to 100 (“worst pain imaginable/so difficult required help”) [49]. Scapular dyskinesis test: scapular position and motion were evaluated, in standing position, at rest and during shoulder abduction and lowering movements [50]. Based on visual observation, a trained physiotherapist classified each participant’s scapular presentation with a type of scapular dyskinesis (intratester reliability of k = 0.49–0.59 [51]) as: (1) type I—when the scapula’s inferior angle was posteriorly displaced/prominent [51,52,53,54]; (2) type II—when the scapula’s medial border was prominent [51,52,54]; (3) type III—when there was excessive elevation of the scapula’s superior border and/or excessive/insufficient scapular upward rotation [51,52,54]; and (4) type IV—when there was symmetry between the scapula of the symptomatic side and the contralateral one [53,54].

To record surface electromyographic (EMG) activity and register the H reflex, the Biopac Systems Inc.—MP 160 Workstation™ (Biopac System Inc., Goleta, CA, USA) was used with a sampling frequency of 2000 Hz, input impedance of 100 MΩ, a common mode rejection ratio (CMRR) of 95 dB and a gain of 1000 and an analog-to-digital converter of 12 bits. Data were collected on UT, MT and LT using steel surface electrodes (TSD150, Biopac Systems Inc., Goleta, CA, USA), bipolar configuration, with an 11.4 mm contact area and an inter-electrode distance of 20 mm. To evoke the H reflex and M-wave through nerve stimulation, the constant current stimulator STMISOLA (Biopac System Inc., Santa Barbara, CA, USA) was used. The locations to place the stimulation electrodes were found through a motor point pen (Compex Iberica, Barcelona, Spain), which was later replaced by self-adhesive Ag/AgCl surface electrodes (Lessa, Barcelona, Spain). Acqknowledge^®^ software (version 3.9, from Biopac Systems Inc., Goleta, CA, USA) was used to create and apply custom-written scripts for stimulator control, EMG signal acquisition, filtering and analysis. An auxiliary tool developed in Python (Python Software Foundation, Beaverton, OR, USA) was used to identify and observe the H reflex and M-wave, as well as to extract the parameters of interest.

### 2.3. Positioning of Electrodes for H Reflex and M-Wave Recordings through Electromyography

**Figure 1:**
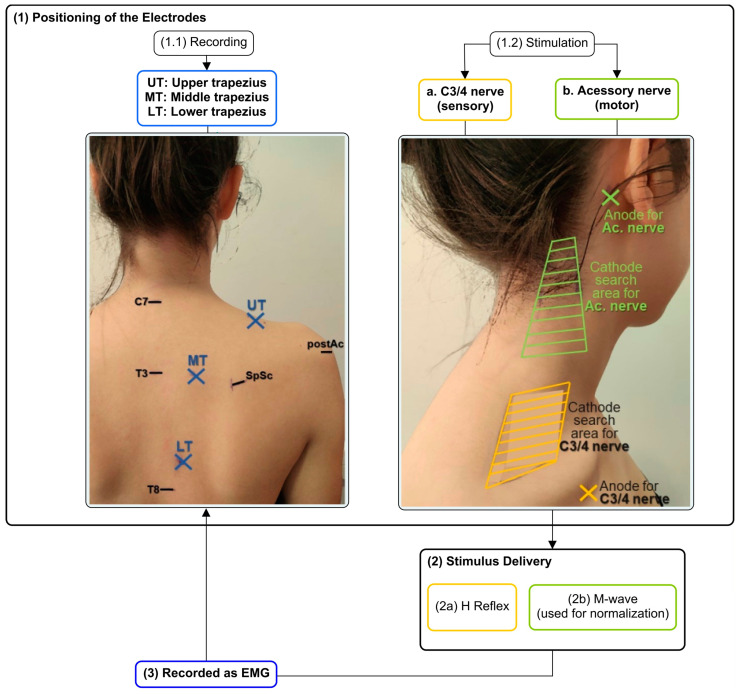
Study protocol steps needed to evoke H reflex and M-wave. Note that: Ac.—accessory nerve; C7—C7 spinous process; postAC—posterior tip of the acromion; SpSc—spine of the scapula; T3—T3 spinous process; T8—T8 spinous process.

To assess the H reflex and M-wave responses of the trapezius muscle, steel surface electrodes were positioned to record EMG from three parts of the trapezius of the painful (or more painful) shoulder. After the skin had been shaved, abraded, and cleaned, ethanol electrodes were placed: (a) UT: over a 2 cm laterally to the midpoint of the line between the spinous process of C7 and the posterior tip of the acromion [55,56]; (b) MT: midway on a horizontal line between the root of the spine of the scapula and the T3 spinous process [19,55]; and (c) LT: obliquely, at 2/3 of the distance along the line from the root of the spine of the scapula to the T8 spinous process [56] (Figure 1). The quality of the raw signals was checked [55,57] before the data were recorded. 

### 2.4. Electrical Stimulation of C3/4 and Accessory Nerves

To allow the stimulation of nerves responsible for the trapezius sensory (C3/4 nerve) and motor (accessory nerve) innervation, the process started with the search for the optimal cathode locations. For this, adhesive electrodes for the anodes were placed just below the middle point of the clavicle [13,30,31,35,39,41,58], or over the mastoid process [13,31,39,58], for the C3/4 nerve or the accessory nerve, respectively. Then, single electrical stimuli (pulse width of 1 ms and intensity of 10 mA) were applied while moving a hand-held motor point pen around the appropriate areas of the neck: (a) around the anterior surface of UT above the clavicle, for C3/4 nerve stimulation [13,30,35,39,41,58]; and (b) behind the sternomastoid muscle and between the level of the jaw and the upper border of the trapezius, for the accessory nerve [13,39,58] (Figure 1). At the locations where the largest responses (H reflex (when C3/4 was stimulated), or M-wave (when accessory nerve was stimulated)) were found, the motor point pen was replaced by self-adhesive electrodes.

During the search for cathode location and also during the process to evoke both H reflex and M-wave, the participants were asked to maintain a sitting position [13,30,31,35,39,41,58,59] with 2/3 of the thigh supported, the knee and hip at 90° of flexion, the feet on the floor [13,31,39,58] and with the head, pelvis and trunk in a neutral position. Both hands were supported on a height-adjustable table, with the shoulder at 90° abduction in the scapular plane, with the forearm in neutral position and the elbow extended. As trapezius H reflex is difficult to elicit at rest [13,31], throughout the H reflex stimulation the same position was adopted but with no support provided to maintain the shoulder position. Therefore, participants maintained the shoulder position through isometric contraction [30,39].

Then, after placing the stimulation and recording electrodes, percutaneous electrical stimulation was delivered separately to the accessory nerve and the C3/4 nerve (Figure 1) by a constant current stimulator controlled with custom-written scripts. The M-wave recruitment curve was evoked by accessory nerve stimulation of 1 ms rectangular pulses [13,35,39,58], 10 s apart, with gradually increased (0.5 mA steps) intensity [13,31,39]. Once the M-wave seemed to be quite stable [34], only one electrical pulse was delivered at each intensity. Recordings were made with the subject relaxed [13,31,39,58] and until there was no further increase in M-wave amplitude in each of the three-trapezius portions in 3 consecutive stimulations, despite an increase in stimulus intensity [13,31,39]. The maximal M-wave (Mmax) was used to normalize the amplitude of the H reflex [13].

C3/4 stimulation was delivered to collect H reflex recruitment curves. Given reflex variability [59], sets of 10 electrical monophasic positive rectangular pulses [30,31,39,41] of 1 ms [13,35,39,58] were delivered with a 10 s interval [60], for each intensity. The first set started with the lowest intensity capable of evoking an H reflex, which was identified by a sequence of single impulses of increasing intensity and at 5 s apart. Then, the intensity was gradually increased by 0.2 mA steps until no further increase in H reflex amplitude, of any trapezius portion, was detected, despite increasing stimulus intensity [13,31,39]. 

### 2.5. Maximal Voluntary Isometric Contractions (MVICs)

At the end of the protocol, the maximal voluntary isometric contractions (MVICs) of each trapezius portion were assessed to normalize the EMG activity level that was produced during the isometric submaximal contraction needed to evoke the H-reflex [UT: 23.0 ± 14.1% and 24.3 ± 14.3% of MVIC; MT: 8.9 ± 12.1% and 11.6 ± 10.7% of MVIC; LT: 13.9 ± 8.4% and 19.0 ± 10.1% of MVIC, for symptomatic and asymptomatic group, respectively]. Each MVIC test was based on the recommendations of Cools et al. [55] and Ekstrom, Soderberg and Donatelli [61]: (a) for UT: shoulder abduction at 90° with the neck in same-side inclination, opposite-side rotation and extension against manual resistance applied at the head and above the elbow, with the subject seated [61]; (b) for MT: shoulder horizontal abduction and external rotation against manual resistance applied above the elbow, with the subject prone [61]; and (c) for LT: shoulder abduction (diagonally at 135°) against a manual resistance, applied with the subject prone [55,61]. Three trials of 5 s MVIC were performed against manual resistance, with 1 min of rest [39] between repetitions to minimize fatigue [62,63]. The 3 central seconds of each contraction were considered, and the mean EMG was calculated.

### 2.6. Data Processing

**Figure 2:**
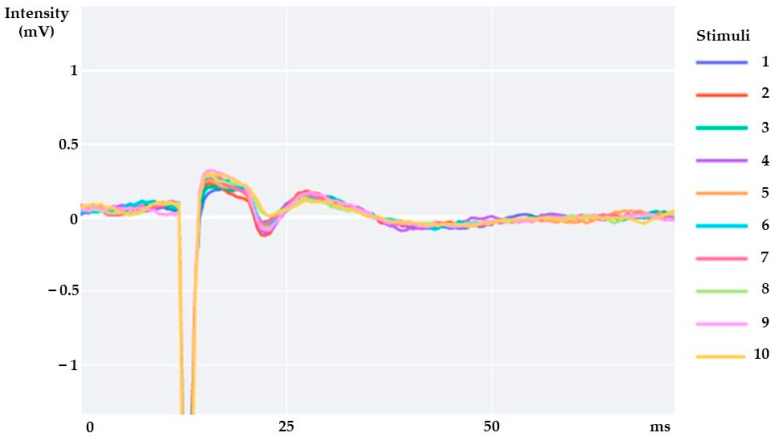
Overlaid traces of H reflexes evoked in upper trapezius by 10 stimuli delivered to C3/4 nerve in one symptomatic participant.

**Figure 3:**
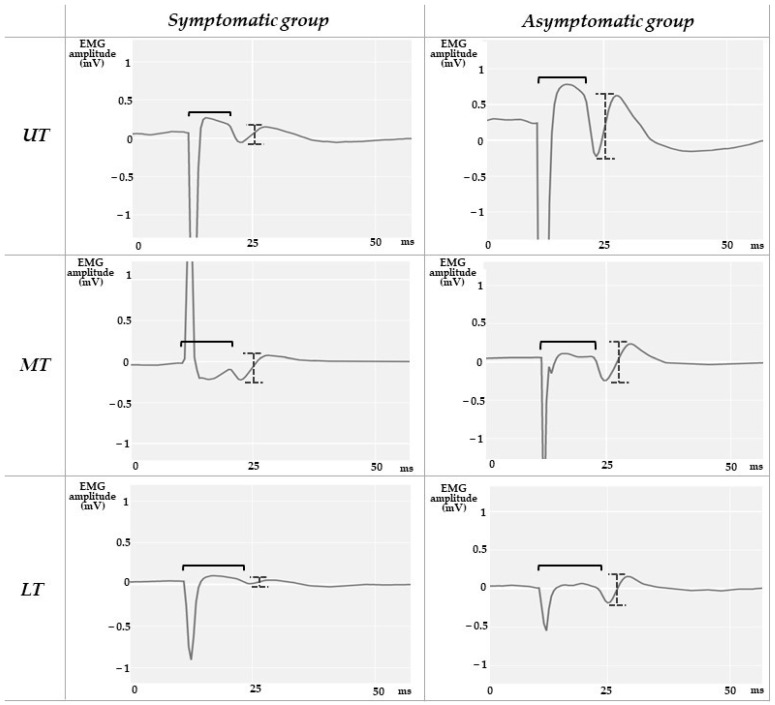
H reflex representation—average of ten reflex responses evoked at the Hmax intensity in three-trapezius portions of an asymptomatic participant and two symptomatic participants with chronic shoulder pain (the UT and MT responses are from one symptomatic participant, while the LT is from another, as none of the symptomatic subjects presented the H reflex in the all the trapezius portions). The black brace represents the latency measurement, while the gray and dotted brace represents the peak-to-peak amplitude of the H reflex.

**Figure 4:**
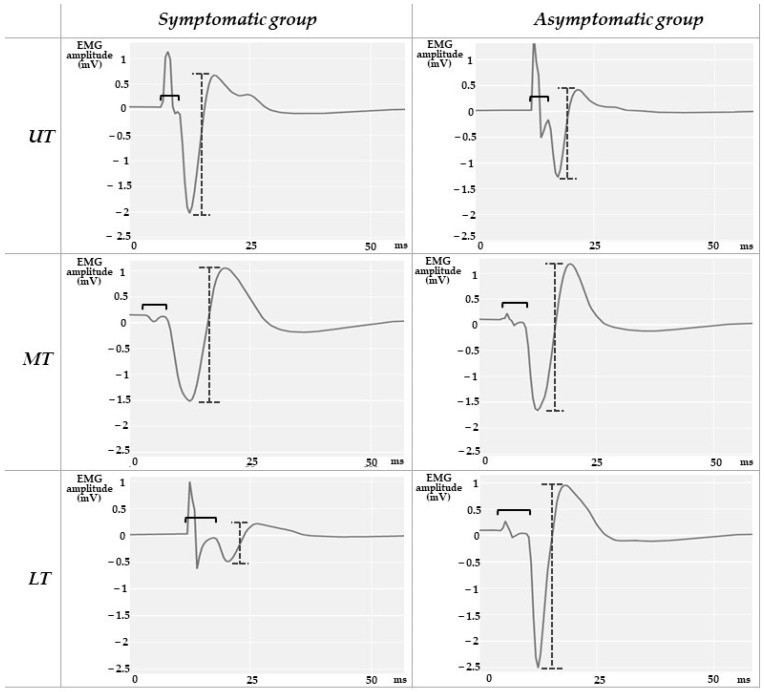
M-wave—representation of the three-trapezius portion in one asymptomatic subject and one symptomatic subject with chronic shoulder pain: black brace represents the latency measurement while the gray and dotted brace represents the peak-to-peak amplitude of the M-wave.

The EMG signals were digitally filtered with a second-order band-pass Butterworth filter with cut-off frequencies ranging between 20 and 1000 Hz. Since for the H reflex 10 stimuli were delivered at each intensity, the 10 responses were overlaid as represented in Figure 2, and averaged for UT, MT, and LT. The latencies of H reflex and M-wave were measured from the stimulus artifact to the onset of the potential. The peak-to-peak amplitudes of H reflex and M-wave were calculated through the mathematical difference between the maximum and minimum values of the compound muscle action potential, for each trapezius portion (Figure 3 and Figure 4). While the maximum value of H reflex peak-to-peak amplitude (Hmax) presents information about the maximum number of motor units reflexively activated [13,38], the maximum peak-to-peak amplitude of M wave (Mmax) represents the maximum motor response [38]. UT, MT, and LT Hmax/Mmax amplitude ratios were calculated for each subject.

### 2.7. Statistical Analysis

The sample size was calculated with G*Power software (Kiel University, Germany) using an effect size (d = 1.218) determined from the difference in latency of a reflex in the lower trapezius found previously between healthy subjects and subjects presenting non-traumatic shoulder instability [29]. It was found that a sample of 10 individuals per group was sufficient to detect differences with a power of 0.8 and an alpha of 0.05. To account for a possible inability to measure the H reflex [13], 12 subjects were included in each group.

Statistical analysis was carried out using the Statistical Package for Social Science (SPSS) version 27 (IBM, Inc., Chicago, IL, USA).

The normality of the data distribution was tested through the Shapiro–Wilk test and histogram analysis. When this assumption was not verified, if possible, a logarithmic or an inverse tangent transformation [64] was applied. Sample homogeneity was tested through the t-student test, Mann–Whitney, or chi-square/Monte Carlo. In all tests, *p* < 0.05 was considered significant. The data were reported as mean/median and standard deviation (SD)/interquartile range (IR) values.

For the comparison of the study variables (the current needed to evoke Hmax (current at Hmax), Hmax, Mmax, the percentage of the ratio between Hmax/Mmax (% Hmax/Mmax) and Mmax and Hmax latencies) between groups, the unpaired-sample *t*-test or the Mann–Whitney test were used. The values of effect size (Cohen’s d) are also presented, except for the variables where a non-parametric test was used. Values higher than 0.8 were considered to represent a large effect size, those of approximately 0.5 a moderate effect size, and those less than 0.2 a small effect size [65].

## 3. Results

Each group contained five male and seven female participants with the demographic characteristics described in Table 1. Groups were well matched at baseline, except for scapular positioning, where the asymptomatic group had only 33% of the subjects presenting some type of scapular dyskinesis while the symptomatic one presented it for 100% of the subjects. Symptomatic subjects also presented a moderate level [46] of shoulder pain (4.42 ± 1.38) and a lower level [49] of functional shoulder disability (17.81 ± 9.02).

### Comparison of Trapezius H Reflex and M-Wave between Symptomatic and Asymptomatic Subjects

**Figure 5:**
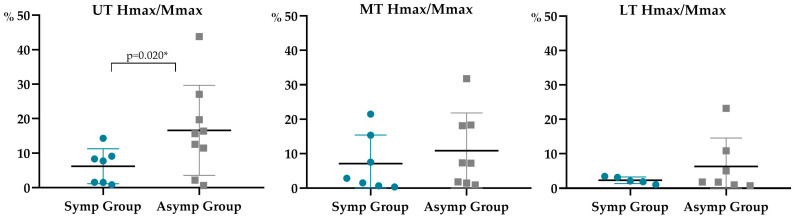
Hmax/Mmax of each participant for different trapezius portions (upper trapezius, UT; middle trapezius, MT; lower trapezius, LT). Hmax is shown as a percentage of Mmax. Symbols represent data for individuals (filled circles, symptomatic; filled squares, asymptomatic). Means and standard deviations are shown as thick horizontal lines with error bars for both groups (symptomatic, Symp group; asymptomatic, Asymp group). Significant results were signed with an *.

**Figure 6:**
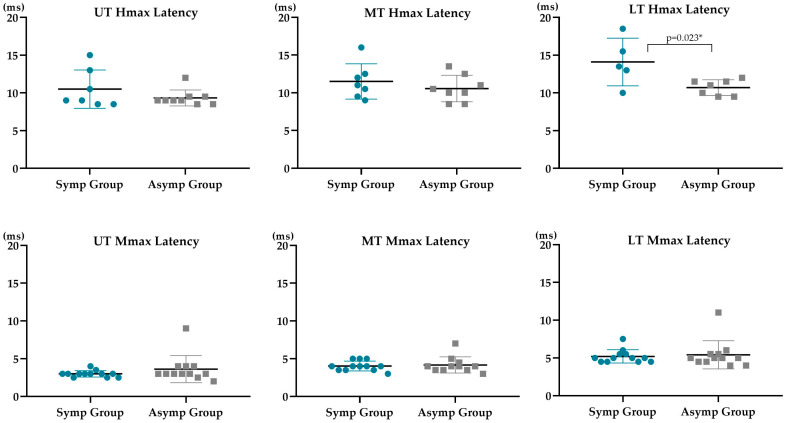
Hmax and Mmax latencies of each participant for different trapezius portions (upper trapezius, UT; middle trapezius, MT; lower trapezius, LT. Symbols represent data for individuals (filled circles, symptomatic; filled squares, asymptomatic). Means and standard deviations are shown as thick horizontal lines with error bars for both groups (symptomatic, Symp group; asymptomatic, Asymp group). Significant results were signed with an *.

Statistically significant differences between groups (*p* = 0.020 and 0.012) were observed for the UT Hmax/Mmax and LT Hmax latency, respectively (Table 2 and Figure 5 and Figure 6). The symptomatic group presented a decreased UT Hmax/Mmax (6.20 ± 5.05%), of less than half of the value for the asymptomatic group (18.58 ± 11.61%), and an increased LT Hmax latency (14.10 ± 3.15 ms), compared with the asymptomatic one (10.71 ± 1.04 ms). In Figure 5, note the low dispersion of UT Hmax/Mmax values in the symptomatic group, while in Figure 6, note the opposite for the LT Hmax latency.

No statistically significant differences (*p* > 0.05) were found between the symptomatic and asymptomatic subjects in % Hmax/Mmax from MT and LT, in Hmax latency from UT and MT, nor in current at Hmax, Hmax, Mmax, and Mmax latency from all trapezius portions (Table 2 and Figure 5 and Figure 6). Moderate effect sizes were observed for the current needed to evoke UT Hmax, the UT Hmax amplitude and UT and MT Hmax latency in the comparisons between groups (Table 2).

## 4. Discussion

Chronic pain has been associated with mechanical and neurophysiological adaptations [66,67,68,69,70,71]. Adaptations in the nervous system [69] have been shown to occur at a supraspinal level [7,29] but also at a spinal level, since muscle pain, mediated by muscle afferents, could alter the muscle spindle discharge and its effect on the motoneuron pool [39]. Therefore, in conditions of pain [33], the H reflex in superficial muscles that have muscle spindles [60] has already been used to give information regarding spinal neurophysiological changes in excitability [5,9,72], pain modulation [31], and integration of peripheral information [9]. The association between the trapezius muscle and musculoskeletal conditions, such as shoulder pain [31], motivated the present study, which compared trapezius spinal excitability between asymptomatic subjects and symptomatic subjects with chronic shoulder pain [58].

Symptomatic subjects reported a moderate shoulder pain intensity (~4.5 out of 10) but a relatively low impact on shoulder function (~18 out of 100), despite all showing scapular dyskinesis. They had significantly lower values of UT Hmax/Mmax than in the asymptomatic group, indicating decreased excitability of the motoneuron pool [35]. Such reduced excitability may suggest the existence of changes in muscle activity during chronic shoulder pain [7], such as a possible decrease in muscular activation [9] from scapula feedback mechanisms [29]. This seems to agree with the findings of previous studies [9,73]. Previous studies have hypothesized decreased activity of the agonist muscles’ motoneurons in muscle pain conditions [73] and that the size of the reflex response is adapted according to the ongoing muscular activity [74]. However, in the present study, the UT ongoing EMG during the recording of the H reflex was 23% and 24% of the maximum, whereas Hmax/Mmax was 6.20% and 18.58% for the symptomatic and asymptomatic groups, respectively. Thus, the impairment in the UT reflex response was not the result of differences in ongoing muscle activity, but rather suggests alterations in the reflex loop. Oliveira Silva et al. [9] have also reported similar results of a decrease in percent Hmax/Mmax from the vastus medialis in subjects with patellofemoral pain, when compared with asymptomatic subjects. Such results confirm the presence of neurophysiological changes in both pain conditions, beyond the structural and biomechanics adaptations [9]. Impairment of the transmission between the Ia afferents and the motoneurons [9,38] could occur at several levels: (a) at the site of stimulation where the afferent volley might be reduced [29,38], (b) presynaptically, where presynaptic inhibition or homosynaptic post-activation depression can alter the neurotransmitter released given the same Ia afferent volley [29,38], and (c) at the motoneurons, through changes in their excitability that makes the Ia input less effective at producing output [7,29,38,75].

In the current study, the symptomatic group also presented increased LT Hmax latency, suggesting a delayed recruitment of the motoneurons through the C3/4 nerve stimulation. One previous study that used stimulation of the ulnar nerve to compare long-latency reflex responses from UT and LT of subjects with shoulder instability and healthy subjects, also reported similar differences between groups for LT latency [29]. Such findings may represent a lower efficiency of the feedback mechanisms acting in scapular stability [29].

Given changes in H reflex parameters in both UT and LT, it may be important to consider whether this could indicate a generalized decrease in trapezius feedback mechanisms [29] and, possibly, a reduction in muscle activation [9]. Although there were no other significant differences between groups, moderate effect sizes were also observed for lengthening of the UT and MT Hmax latencies, (d = −0.918 and −0.505, respectively), and Hmax/Mmax values for MT and LT tended to be lower for the symptomatic group. Together, these non-significant results point in the direction of a generalized effect. Moreover, the lack of results could be due to the reduced sample sizes. The number of participants in whom it was possible to evoke the H reflex was not the same for each group and each part of the trapezius. Thus, it may have limited the identification of significant results. However, it should be noted that, regardless of wider effects, the isolated statistically significant deficits for UT Hmax/Mmax and LT Hmax latency could represent an impact on the function of the shoulder complex, given the possible imbalance among trapezius portions [29]. Reflex motoneuron activation seems to have an impact on scapula stability and, consequently, on upper-limb functionality. Thus, such an imbalance regarding the muscular control of the portions of trapezius could reflect an impairment of the feedback mechanisms that act during scapula motion, as well as differences in the coupling of forces responsible for scapular movement and stability. In consequence, impairments in scapula-humeral rhythm could be found [29]. In the present study, the results regarding scapular positioning assessed at rest and during motion seem to reinforce the hypothesis that impairments at UT Hmax/Mmax and LT Hmax latency could be associated with changes in scapular stability and motion, as all the participants of the symptomatic group had altered scapular positioning, compared to only 34% of the participants of the asymptomatic group.

In the current study, a moderate effect size (d = 0.586) was also observed for the current intensity needed to evoke the UT Hmax. Lower values were observed in the symptomatic group, perhaps because the symptomatic group did not have the ability to increase the H reflex to values equal to the asymptomatic one, noting the moderate effect size for the UT Hmax (d = 0.957). This may be in line with the results of a previous study [38] that evaluated the H reflex in patients with low back pain compared with healthy subjects. In that case, in the group with pain, the H reflex size grew more slowly as the stimulus intensity increased, and the H reflex threshold was higher [38].

In short, impairments in the scapula’s reflex control could compromise its stabilization and, consequently, the function of the shoulder and the whole upper limb [29]. Thus, rehabilitation should also be planned considering this neurophysiological parameter, with the expectation that it could contribute to longer-lasting results. A previous study suggested the use of proprioceptive exercises and sensory stimulation to enhance the H reflex through more inputs [9]. Moreover, in cases of different adaptations between trapezius portions, exercises focusing on scapula motor control and the balance of muscular activity [55,76,77,78] could also be important for subjects suffering from chronic shoulder pain associated with changes in H reflex variables such as Hmax/Mmax. Feedback from wearable sensors could even allow the retraining of scapula control in daily activities [79].

### Limitations

The present study had some limitations. The first one was the long duration of the protocol, given the necessity of searching for a very specific location for the nerve stimulation, together with the recording of 10 stimuli for each intensity to evoke the H reflex (justified given the high variability of the reflex [60]). Such a factor could be associated with higher demands and fatigue, which could affect the scapular muscles’ motor control [80] and the EMG signals [81], and could also limit the applicability and prevent quick access to the evaluation results. However, previous studies have reported inconsistent effects with decreased, increased or unchanged ratios of Hmax/Mmax after fatigue [82]. Therefore, we believe that fatigue likely did not have a very strong influence on the results obtained in the present study. Based on these limitations, the development of tools to facilitate the performance of this measurement in clinical practice is required. Another limitation was the inability to identify an H reflex for the three-trapezius portions of all subjects when the motor point pen was changed for adhesive electrodes, which consequently led to a lower final-sample size. This occurred, even though the H reflex was evoked during an isometric contraction to ensure that the motoneurons were close to their threshold and more easily activated by the Ia afferent volley [58]. However, it was expected, since it also happened in previous studies [13,30,39,58]. Finally, the present study only assessed the reflex response, and the findings do not allow insight into other nervous system structures or other scapular variables to better characterize the origin and/or proportions of the adaptations. Future studies assessing the H reflex, together with variables related to voluntary control or the scapular kinematic and muscular activity levels, could be important.

## 5. Conclusions

Subjects with chronic shoulder pain present decreased UT Hmax as a percentage of Mmax, as well as a longer LT H reflex latency. Despite the moderate effect of size and the same tendencies being observed for the other trapezius portions, a significant finding for only one portion may be important, since it could indicate an imbalance between the activity of these scapular stabilizers.

## Figures and Tables

**Table 1:** Groups’ demographic characteristics and frequency of H reflex and M-wave responses.

		Asymptomatic Group (*n* = 12)	Symptomatic Group (*n* = 12)	Comparison between Groups	
Parameter		mean ± SD	mean ± SD	*t*	*p*-value
Height (m)		1.7 ± 0.09	1.7 ± 0.08	0.345	0.733
Weight (kg)		69.8 ± 14.02	68.3 ± 14.10	0.269	0.791
BMI (kg/m^2^)		23.0 ± 3.12	22.9 ± 3.50	0.127	0.900
		median ± IR	median ± IR	*U*	*p*-value
Age (years)		23.0 ± 9.00	22.0 ± 2.75	58.00	0.443
		frequency (*n*)	frequency (*n*)	Pearson X2	*p*-value
Scapular positioning	Without changes	67% (*n* = 8)	0% (*n* = 0)	12.000	0.001 *
	Presenting a dyskinesis type	33% (*n* = 4): Two subjects with type II and two with type III	100% (*n* = 12): Three subjects with type I, two with type II, four with type III and two with type II + III		
H reflex—frequency of responses identified	UT	75% (*n* = 9)	58% (*n* = 7)	0.750	0.386
	MT	75% (*n* = 9)	58% (*n* = 7)	0.750	0.386
	LT	58% (*n* = 7)	42% (*n* = 5)	0.667	0.414
M-wave—frequency of responses identified	UT	100% (*n* = 12)	100% (*n* = 12)	-	-
	MT	92% (*n* = 11)	100% (*n* = 12)	1.043	1.000
	LT	100% (*n* = 12)	100% (*n* = 12)	-	-

Footnote—Missing data occurred for H reflexes (indicated by response frequency) when it was not possible to identify the H reflex after application of the adhesive electrodes or when, instead of an H reflex, an M-wave response (identified through its latency) occurred during the C3/4 nerve stimulation, possibly due to the stimulus spreading or the anatomical variation of the C3/4 nerve [58]. Significant results were signed with an *.

**Table 2:** H reflex and M-wave variables.

		Asymptomatic Group	Symptomatic Group	Comparison between Groups		Effect Size	
Parameter	Muscle	mean ± SD	mean ± SD	*t*	*p*-value		
Current at Hmax (mA)	UT	5.83 ± 1.28	4.75 ± 2.41	1.163	0.264	0.586	
	MT	5.50 ± 1.34	6.79 ± 5.90	0.448	0.661	0.226	
	LT	4.79 ± 1.85	5.68 ± 2.95	−0.648	0.532	−0.379	
Hmax (mV)	UT	0.34 ± 0.28	0.13 ± 0.10	1.628	0.060	0.957	
	MT	0.14 ± 0.21	0.15 ± 0.17	−0.053	0.959	−0.027	
	LT	0.12 ± 0.16	0.04 ± 0.02	0.503	0.630	0.252	
Mmax (mV)	UT	1.64 ± 0.99	2.20 ± 1.48	−1.076	0.293	−0.439	
	MT	1.83 ± 1.38	1.90 ± 0.93	−0.159	0.875	−0.066	
	LT	2.02 ± 1.10	1.84 ± 0.66	0.494	0.626	0.202	
% Hmax/Mmax	UT	18.58 ± 11.61	6.20 ± 5.05	2.619	0.020 *	1.320	
	MT	10.86 ± 10.95	7.10 ± 8.28	0.741	0.472	0.383	
	LT	6.33 ± 8.25	2.32 ± 0.96	0.701	0.503	0.357	
Hmax latency (ms)	UT	8.94 ± 0.39	10.50 ± 2.55	−1.600	0.159	−0.918	
	MT	10.50 ± 1.66	11.50 ± 2.35	−1.001	0. 334	−0.505	
	LT	10.71 ± 1.04	14.10 ± 3.15	−2.692	0.023 *	−1.576	
				*U*	*p*-value		
Mmax latency (ms)	UT	3.62 ± 1.80	3.00 ± 0.43	57.000	0.343	-	
	MT	4.18 ± 1.08	4.04 ± 0.66	66.500	0.974	-	
	LT	5.42 ± 1.86	5.21 ± 0.86	75.500	0.835	-	

Footnote—Significant results were signed with an *. Note that, for each H Reflex or M-wave parameter, the number of subjects included in the mean ± SD is reported in Table 1 as “frequency of responses identified”. For %Hmax/Mmax, only the participants that presented both H Reflex and M-wave responses were considered.

## Data Availability

Not applicable.
